# Supplementary material for: Functionalized Boron Nitride Nanosheets/Poly(l-lactide) Nanocomposites and Their Crystallization Behavior
Source: Polymers (Basel). 2019 Mar 6;11(3):440. doi: 10.3390/polym11030440 (PMC6473543; doi:10.3390/polym11030440)
Supplement: Supplementary file 1 [file polymers-11-00440-s001.pdf]

Supplementary materials

Functionalized Boron Nitride Nanosheets/Poly(L-lactide) Nanocomposites and Their Crystallization Behavior

Deyu Kong [1], Deli Zhang [1], Hongge Guo [1], Jian Zhao [1,2,3,*], Zhaobo Wang [1], Haiqing Hu [1,*], Junting Xu [4,*] and Cuiliu Fu [1,2]

[1] Key Laboratory of Rubber-Plastics Ministry of Education/Shandong Provincial Key Laboratory of Rubber-Plastics, Qingdao University of Science & Technology, No. 53 Zhengzhou Road, Qingdao 266042, China; and School of Materials Science and Engineering, Qilu University of Technology (Shandong Academy of Sciences), Jinan 250353, China; kdy0511@163.com (D.K.); zhangdl513@163.com (D.Z.); 921925576@qq.com (H.G.) ; wangzhb@qust.edu.cn (Z.W.); clfu@ciac.jl.cn (C.F.)

[2] State Key Laboratory of Molecular Engineering of Polymers, Fudan University, Shanghai 200433, China

[3] Key Laboratory of Polymer Processing Engineering (South China University of Technology), Ministry of Education, Guangzhou 510640, China

[4] MOE Key Laboratory of Macromolecular Synthesis and Functionalization, Department of Polymer Science and Engineering, Zhejiang University, Hangzhou 310027, China

* Correspondence: zhaojian@qust.edu.cn (J.Z.); hhq@qust.edu.cn (H.H.); xujt@zju.edu.cn (J.X.); Tel.: +86-532-84-022-725 (J.Z.); +86-571-8795-3164 (J.X.); Fax: +86-571-87-952-400 (J.X.)

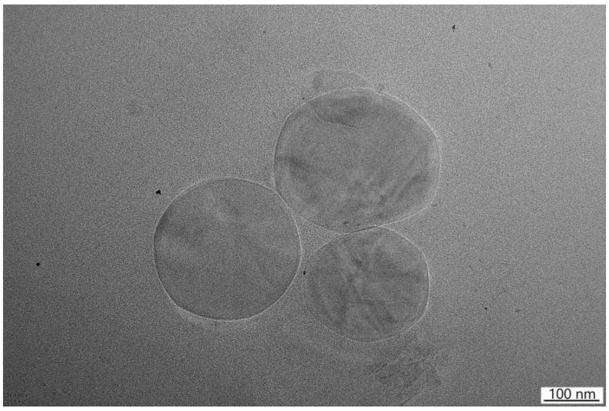

Figure S1. TEM image of hydroxyl-functionalized boron nitride nanosheets (OH-BNNS) .

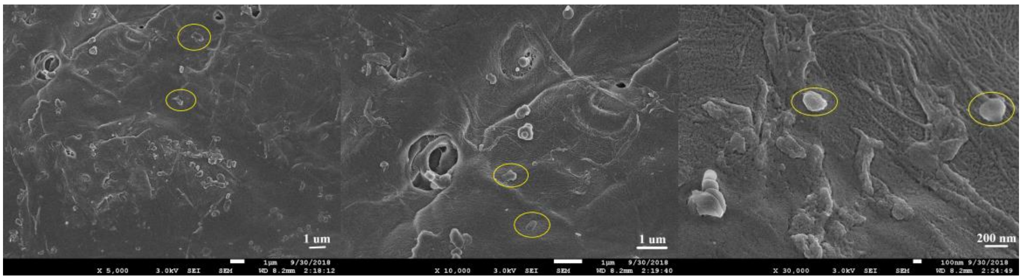

Figure 2. SEM images of the PLLA nanocomposite(with the OH-BNNS loading of 1wt %).

To characterize the morphology and dispersion state of OH-BNNS in the nanocomposite, the PLLA nanocomposite (1wt %) were examined by SEM. As displayed in Figure S2, OH-BNNS nanoplatelets were well separated in the matrix and no noticeable OH-BNNS aggregation was observed . This reflected good compatibility of OH-BNNS with the PLLA matrix.

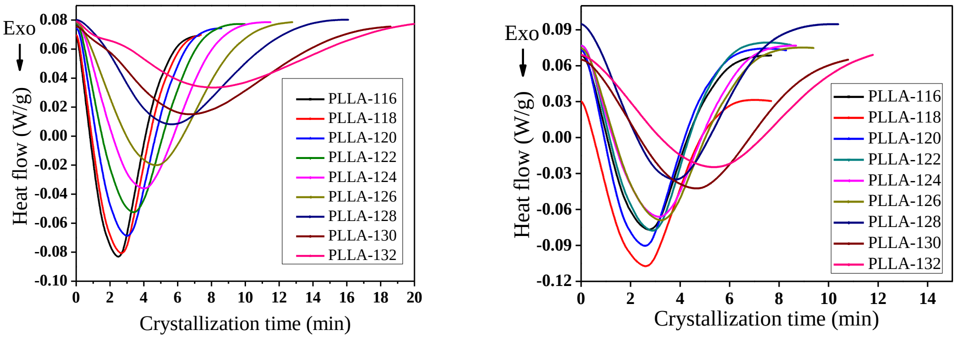

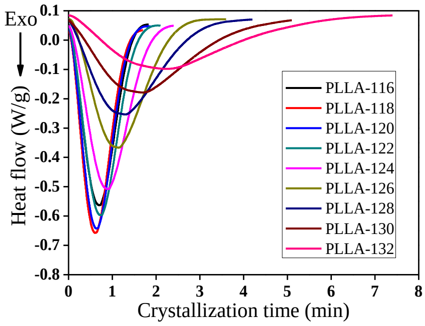

Figure S3. Heat flow curves of PLLA-0.5 (a) PLLA-1 (b) and PLLA-3 (c) during isothermal cold crystallization at different crystallization temperatures.

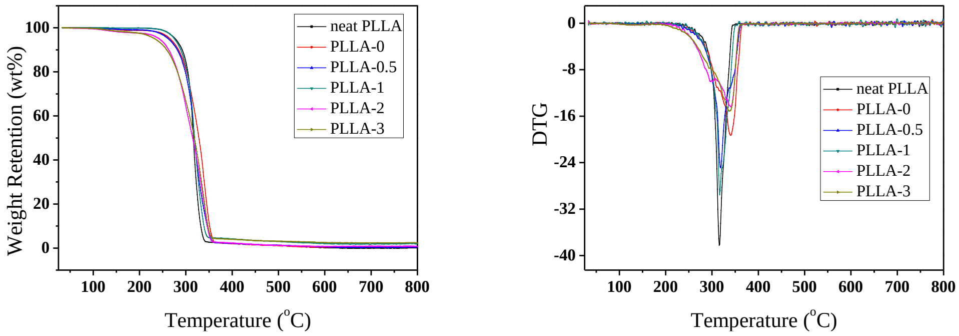

Figure S4. The TGA (a) / DTG (b) curves of neat PLLA and PLLA/OH-BNNS nanocomposites.

The weight loss curves upon heating and corresponding differential curves are shown in Figures S4 (a) and (b), respectively. The residual weight percentages at 700 °C are 0.42, 0.90, 1.80 and 2.51 wt% for PLLA-0.5, PLLA-1, PLLA-2 and PLLA-3, respectively.
